# Supplementary material for: Semi-supervised segmentation of RNA 3D structures using density-based clustering
Source: Comput Struct Biotechnol J. 2025 Sep 10;27:3966–84. doi: 10.1016/j.csbj.2025.08.037 (PMC12800371; doi:10.1016/j.csbj.2025.08.037)
Supplement: Supplementary file 1 — Supplementary material [file mmc1.pdf]

Supplementary figures

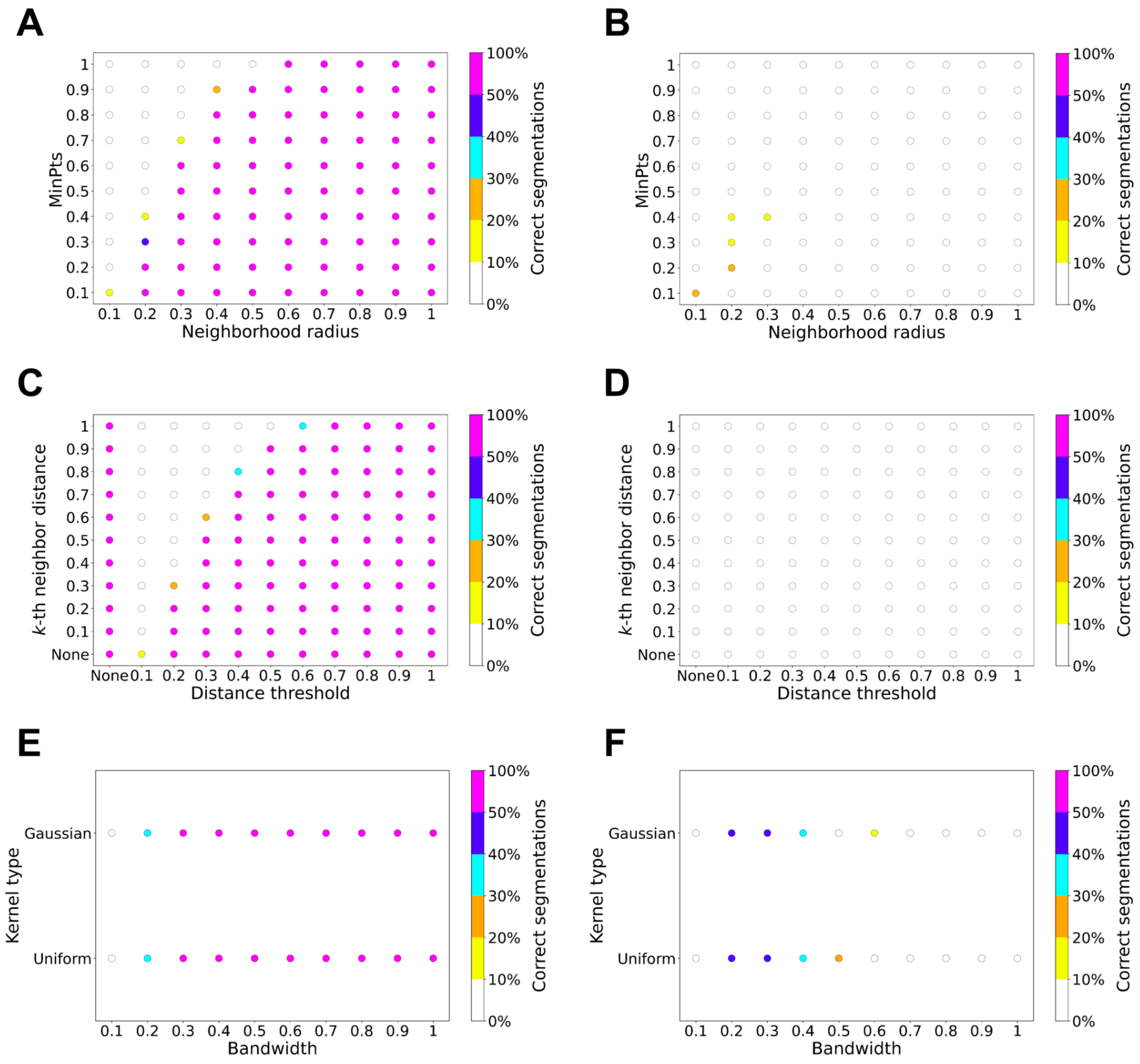

Figure S1. Scatter plots representing the search for hyperparameter values that return a correct segmentation in terms of number of domains, for (**A**, **B**) DBSCAN, (**C**, **D**) HDBSCAN and the hybrid approach, and (**E**, **F**) Mean Shift. The computations were performed on the 26 experimental RNAs structures from the RNA3DB set: (**Left panels**) The subset of 16 single-domain annotations; (**Right panels**) The subset of 10 multi-domain annotations. The color of each dot represents the percentage of cases with the right number of clusters, as shown by the color scale.

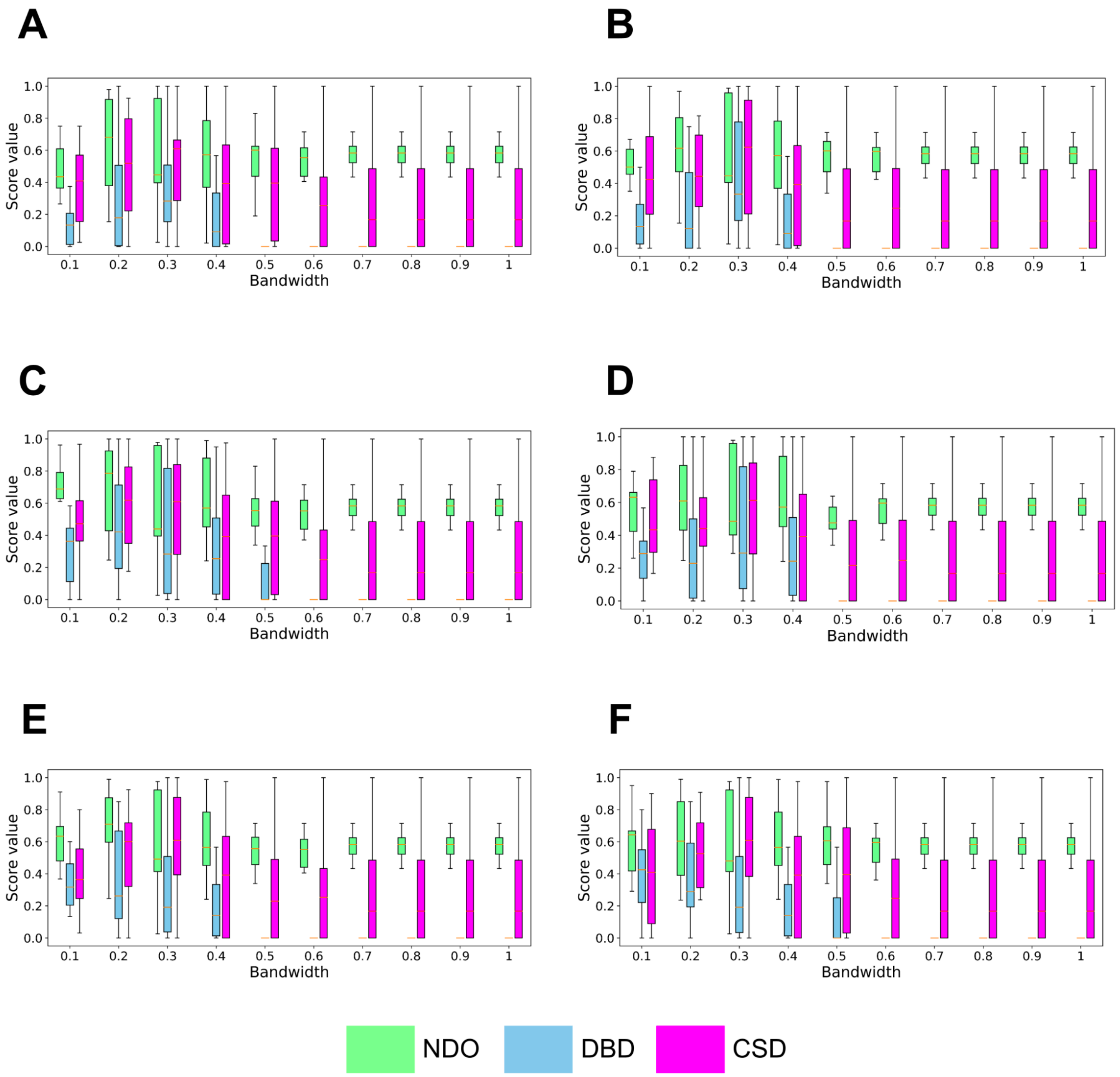

Figure S2. The NDO, DBD, and CSD values obtained with the Mean Shift clustering, using different hyperparameter values calculated on the subset of multi-domain RNA structures (n=10 entries) from the RNA3DB set. For the bandwidth, 10 values were tested (expressed as a ratio of quantile). Atomic representation and kernel type: (**A**) C1’-only + uniform kernel; (**B**) C1’-only + Gaussian kernel; (**C**) C3’-only + uniform kernel; (**D**) C3’-only + Gaussian kernel; (**E**) C4’-only + uniform kernel; (**F**) C4’-only + Gaussian kernel.

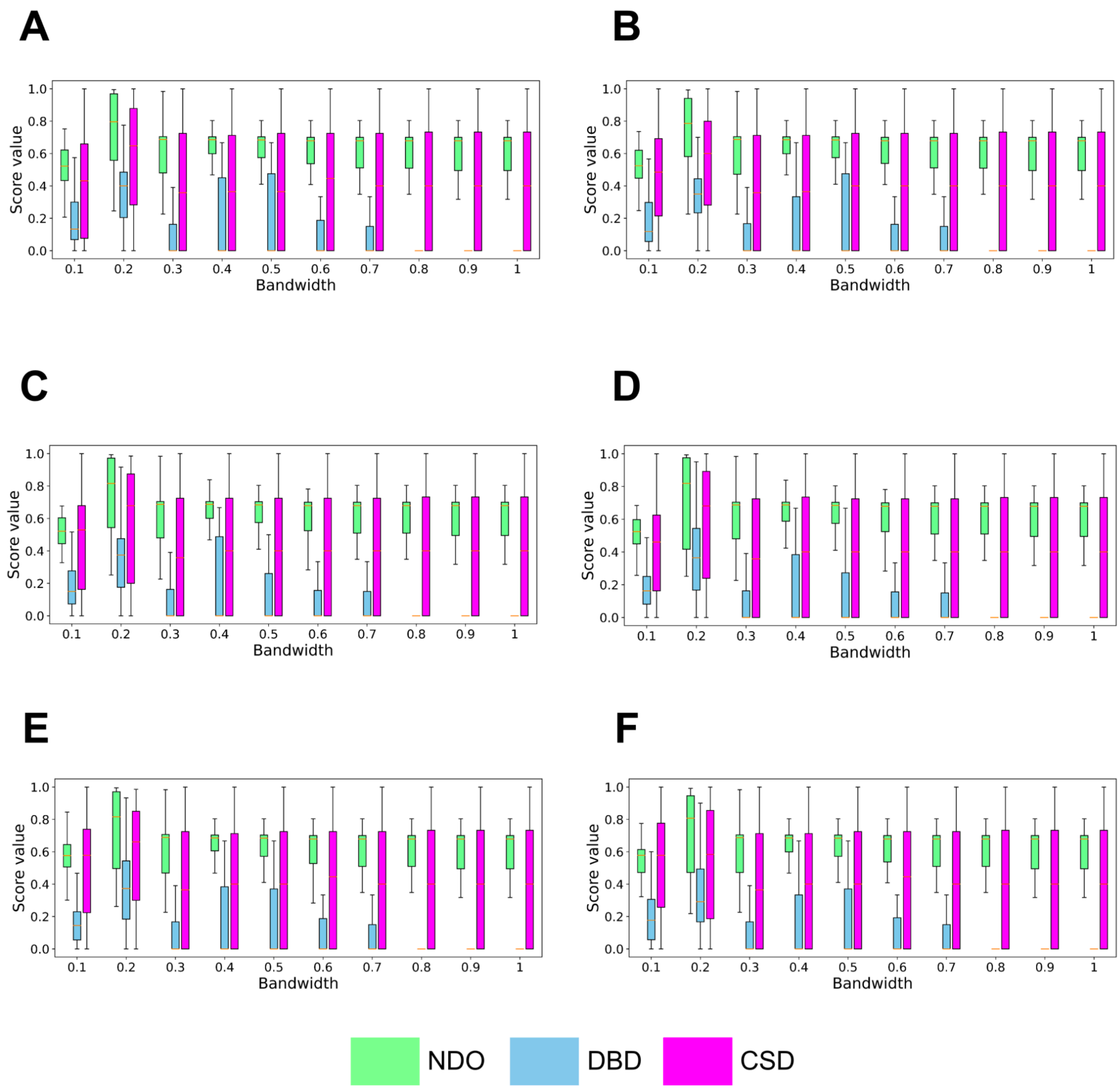

Figure S3. The NDO, DBD, and CSD values obtained with the Mean Shift clustering, using different hyperparameter values calculated on the subset of multi-domain RNA structures (*n*=50 entries) from the RNA3DHub set. For the bandwidth, 10 values were tested (expressed as a ratio of quantile). Atomic representation and kernel type: (**A**) C1'-only + uniform kernel; (**B**) C1'-only + Gaussian kernel; (**C**) C3'-only + uniform kernel; (**D**) C3'-only + Gaussian kernel; (**E**) C4'-only + uniform kernel; (**F**) C4'-only + Gaussian kernel.

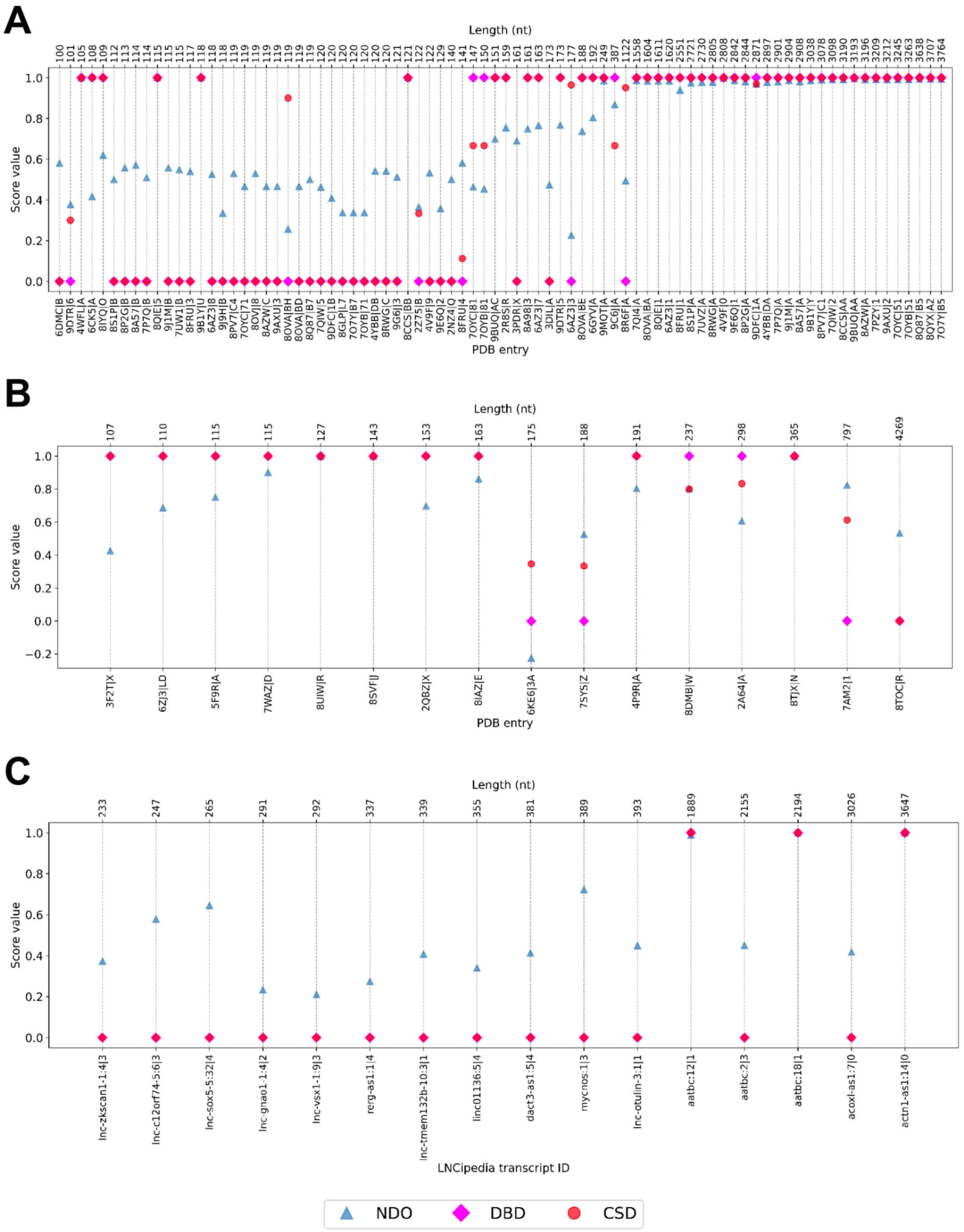

Figure S4. The NDO, DBD, and CSD values obtained for the single-domain RNA 3D structures from (**A**) RNA3DHub, (**B**) RNA3DB and (**C**) LNCipedia dataset. The samples are sorted by ascending RNA length, from left to right of the figure. Detailed score values are provided in Tables S6 to S8.
